# Cardiac Contractile Reserve Parameters Are Related to Prognosis in Septic Shock

**DOI:** 10.1155/2013/930673

**Published:** 2013-07-17

**Authors:** Antoine Kimmoun, Nicolas Ducrocq, Sébastien Mory, Remi Delfosse, Laura Muller, Pierre Perez, Renaud Fay, Bruno Levy

**Affiliations:** ^1^Service de Réanimation Médicale Brabois, CHU de Nancy, 54511 Vandoeuvre les Nancy, France; ^2^INSERM, Centre d'Investigations Cliniques-9501 and CHU de Nancy, 54511 Vandoeuvre les Nancy, France; ^3^Université de Lorraine, Nancy, France

## Abstract

*Introduction.* Cardiac reserve could be defined as the spontaneous magnitude from basal to maximal cardiac power under stress conditions. The aim of this study was to evaluate the prognostic value of cardiac reserve parameters in resuscitated septic shock patients. *Methods*. Seventy patients with septic shock were included in a prospective and observational study. Prior to inclusion, patients were resuscitated to reach a mean arterial pressure of 65–75 mmHg with an euvolemic status. General, hemodynamic, and cardiac reserve-related parameters (cardiac index, double product, and cardiac power index) were collected at inclusion and at day 1. *Results*. Seventy patients were included with 28-day mortality at 38.5%. Ten of the 70 patients died during the first day. In multivariate analysis, independent predictors of death were SAPS II ≥58 (OR: 3.36 [1.11–10.17]; *P* = 0.032), a high double product at inclusion (OR [95% IC]: 1.20 [1.00–1.45] per 10^3^ mmHg*·*min; *P* = 0.047), and at day 1, a decrease in cardiac index (1.30 [1.08–1.56] per 0.5 L/min/m^2^; *P* = 0.007) or cardiac power index (1.84 [1.18–2.87] per 0.1 W/m^2^, *P* = 0.008). *Conclusion*. In the first 24 hours, parameters related to cardiac reserve, such as double product and cardiac index evolution, provide crucial and easy to achieve hemodynamic physiological information, which may impact the outcome.

## 1. Introduction

Myocardial dysfunction is a frequent (30%–70%) complication during septic shock characterized by a biventricular systolic and diastolic dysfunction [1]. Usually, in septic shock patients, meeting the increased metabolic demand requires a rise in oxygen transport. In fluid-resuscitated septic shock patients, cardiac output is generally elevated due to decreased systemic vascular resistances but may be insufficient to meet the increased oxygen demand and could therefore impact outcome. However, it is very difficult to establish whether septic cardiomyopathy independently affects the prognosis of patients with septic shock. Vieillard-Baron et al. found that prognosis is poor in the presence of a hyperkinetic state, which reflects persistent and profound vasoplegia [2]. The severe reduction of afterload observed in septic shock may often mask cardiac impairment, enabling a severely diseased heart to pump a seemingly “normal” cardiac output. Nevertheless, in the case of a severely depressed systemic vascular resistance of 200–300 dynes, cardiac output should be as high as 15–20 L/min to maintain arterial blood pressure [3]. Cardiac contractile reserve (CCR) is one means to assess the efficiency of cardiac adaptation to septic shock. In physiology, CCR could be defined as the spontaneous magnitude from basal to maximal cardiac power output and/or its responsiveness under stress test [4]. The work performed at each cardiac cycle to eject blood defines the stroke work. The product of stroke volume and mean arterial pressure allows an estimation of stroke work. By extension, cardiac power output used to assess CCR is defined by the product of stroke work and heart rate [5]. In critically ill patients, many CCR-related parameters (oxygen consumption, stroke work, cardiac power …) have been studied, most of which are derived from cardiac index (CI) magnitude during a dobutamine test [6]. Double product (systolic arterial pressure × heart rate) is an old hemodynamic tool, closely related to CR and known to have low values in chronic heart failure [7–9]. Its evolutive pattern has never been established during shock states.

Until now, no study has assessed the evolution of CCR-related parameters without the use of a pharmacological stress test in order to predict outcome in the setting of current guidelines [10]. We hypothesized that (i) septic shock itself is a sufficient stress condition to test CCR during septic shock and (ii) both the importance of vasoplegia and cardiac adaptation are prognosis factors of septic shock evolution. Hence, the present report is a prospective study aimed at evaluating the predictive value of CCR parameters on mortality rate in septic shock patients treated according to Surviving Sepsis Campaign recommendations.

## 2. Methods

### 2.1. Patients

The study was conducted in a 13-bed medical Intensive Care Unit of a University Hospital. Patients were included in the first twelve hours after the diagnosis of septic shock defined by a systolic arterial pressure (SAP) <90 mmHg (or a decrease >50 mmHg in patients known to be hypertensive), and persisting mean arterial pressure (MAP) <70 mmHg or diastolic arterial pressure (DAP) ≤40 mmHg despite adequate fluid resuscitation requiring vasoactive support by norepinephrine (>0.05 *μ*/kg/min) during more than one hour. Resuscitation followed the Surviving Sepsis Campaign guidelines for the management of septic shock patients. Norepinephrine was initiated at 0.2 *μ*g/kg/min, and infusion rate was rapidly increased and adjusted to maintain an MAP of 65–75 mmHg. Optimization of volemia before inclusion and throughout the entire inclusion period was confirmed by the following fluid responsiveness-prediction tests: delta pulse pressure (PP) <12%, increase in cardiac index (CI) <10% during a passive leg raising test and/or increase <5% in PP during the end-expiratory occlusion test. Strategy included at least two fluid responsiveness-prediction tests performed every three hours or when deemed necessary according to the physician in charge. Any discordance between these two tests was followed by a 500 mL fluid challenge. An echocardiography was also performed to assess left ventricular fraction and respiratory variation of the inferior vena cava (diameter max − diameter min)/((diameter max + diameter min)/2) which must be <12% before inclusion. Particular attention was given to the normalization of the initial ScVO_2_ and lactatemia. When CI was <2.5 L/min/m^2^ or/and ScVO_2_ <70% despite optimization of volemia at an MAP maintained between 65–75 mmHg, dobutamine was initiated at 5 *μ*g/kg/min and then adapted to CI and ScVO_2_. Antibiotics were also administered as soon as possible and adapted to the putative infection site. Hydrocortisone (50 mg × 4/day) was considered when patients remained vasopressor-dependent after at least six hours of norepinephrine therapy. This observational study was approved by our local and institutional Ethics Committees (Comité de Réflexion Ethique Nancéien Hospitalo-Universitaire). This observational and noninterventional study did not require any consent. An information letter was delivered to each patient (or patient's representative) included in this study.

### 2.2. Measurements

Patients were monitored using a PiCCO 2 device (Pulsion Medical Systems, Munich, Germany) connected to a central venous jugular catheter and a thermistor-tipped arterial catheter in the femoral artery (PV2015L20N, Pulsion Medical Systems, Munich, Germany). The arterial catheter was connected to a specific PV8215 pressure sensor using a PV8215 monitoring kit (Pulsion Medical Systems, Munich, Germany). The average of three thermodilutions was systematically achieved during a hemodynamically stable period to obtain CCR-related parameters derived from the PiCCO device: CI, cardiac power index (CPI = CI × MAP/451), double product (DP = SAP × Heart Rate [HR]), left ejection fraction (LEF), global end diastolic volume (GEDV). Pulse contour derived cardiac output was continuously recorded on the PiCCO 2 device. Indexed systemic vascular resistance (ISVR) was calculated from MAP, CI, and measurement of central venous pressure (CVP) by a standard pressure transducer connected to the central venous jugular catheter (ISVR = [MAP – CVP]/CI × 80).

Biological analyses were performed in parallel: blood gases, central venous oxygen saturation (ScVO_2_), creatinine, hemoglobin, platelets, leukocytes, prothrombin, B-type natriuretic peptide, troponin T, and procalcitonin.

### 2.3. Study Design

After patient optimization defining “day 0,” a first set of hemodynamic measurements and biological analyses were performed. Twenty-four hours after inclusion defining “day 1,” the same above hemodynamic and biological data were collected. Duration of catecholamine infusion, length of hospital stay, and mortality at 28 days were also recorded.

## 3. Statistical Methods

All analyses were performed using SAS software R9.3 (SAS Institute, Cary, NC, USA). The two-tailed significance level was set at *P* < 0.05. Continuous variables are presented as mean ± standard deviation (m ± SD) and categorical variables as frequency (percent). Univariate comparisons were carried out using the Mann-Whitney or Chi-square test, as appropriate. Association of baseline characteristics (70 patients) and 1-day change from baseline (60 patients) with mortality were assessed in three separate models using multivariate logistic regression: baseline factors were tested in 2 models with and without SAPS II since this latter composite score will expectedly be strongly associated with prognosis and could mask other important factors; change at day 1 was analyzed in a 3rd model in 24 h survivors. All factors listed in Table 2 were tested in these three separate models. The final models retained only significant factors. Linearity of the association between factors and mortality (relationship between the independent and dependent variables which must be linear) was assessed by plotting the regression coefficients against the midpoints of tertiles of distribution. SAPS II, whose effect cannot be considered as linear, was dichotomized according to the median (SAPS II ≥58) as the best balance between sensitivity and specificity using ROC curves. Other validity assumptions of the models (absence of interaction and collinearity, goodness-of-fit) were thoroughly verified. Odds ratios and event rates are illustrated, respectively, by forest plots and Kaplan-Meyer curves. Thresholds for Kaplan-Meyer plots were identified using ROC curves as above.

## 4. Results

### 4.1. Population Characteristics

Seventy consecutive patients with septic shock were included (19 women, 51 men, mean age 62 ± 16 years) among whom 43 (61.4%) were alive at 28 days. Ten patients died before 24 hours. Patient characteristics (medical history, bacterial source, site of infection) are summarized in Table 1.

### 4.2. Day 0 Characteristics (Table 2)

**Figure 1:**
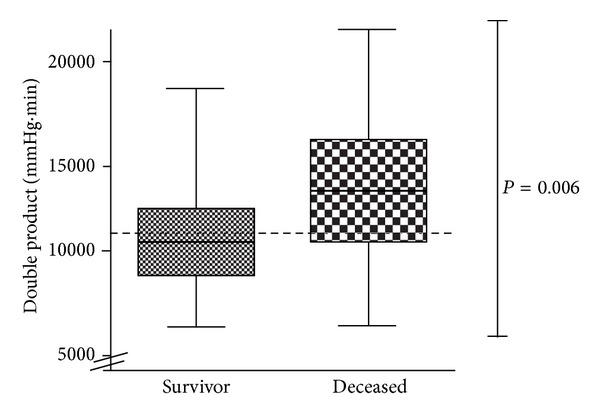
Double Product at day 0 in survivors and nonsurvivors. Dashed line: cut-off at 11000 mmHg/s.

In univariate analysis, nonsurvivors had a significantly higher CI, HR, DP, and SAPS II (CI: 3.91 ± 1.35  *versus*  3.28 ± 1.18 L/min/m^2^; *P* = 0.036, HR: 115 ± 26  *versus*  97 ± 23 bpm; *P* = 0.07, DP: 13310 ± 3385   *versus*   10990 ± 2847 mmHg·bpm (Figure 1); *P* = 0.006 and SAPS II: 53 ± 16  *versus *69 ± 22; *P* = 0.005).

**Figure 2:**
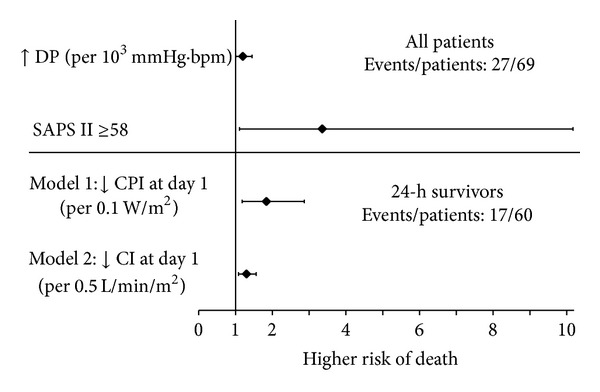
Multivariate analysis for Double Product (DP), SAPS II, Cardiac Index (CI) and Cardiac Power Index (CPI). bpm, beats per minute; SAPS II, Simplified Acute Physiology Score.

In multivariate analysis, a high DP (OR 1.20 [1.00–1.45] per 10^3^ mmHg·bpm; *P* = 0.047) and SAPS II ≥58 (OR: 3.36 [1.11–10.17]; *P* = 0.032) were the only two factors associated with 28-day mortality (Figure 2). 

Biological parameters were also associated with mortality at 28 days in univariate analysis (online appendix; see Supplementary Material available online at http://dx.doi.org/10.1155/2013/930673): nonsurvivors had lower pH (7.27 ± 0.14  *versus*  7.34 ± 0.09; *P* = 0.022), lower hemoglobin levels (9.2 ± 1.7  *versus*  10.5 ± 1.8 g/dL; *P* = 0.005), and lower platelet levels (152 ± 158  *versus*  241 ± 170 G/l; *P* = 0.01). 

### 4.3. Twenty-Four Hour Change from Day 0 (Table 2)

At day 1, except for platelet levels (nonsurvivors: 132 ± 108 G/l  *versus*  survivors: 211 ± 123 G/l; *P* = 0.04), no differences in hemodynamic or biological data were found between 28-day survivors and nonsurvivors (Table 2).

**Figure 3:**
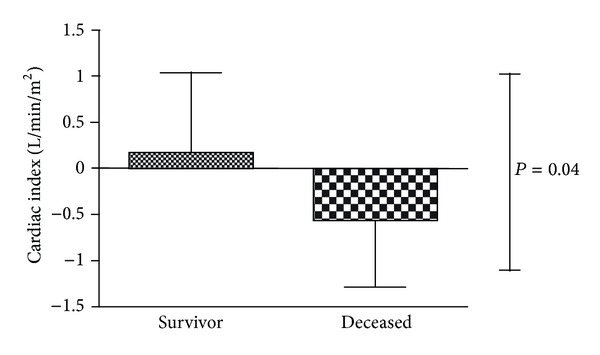
Cardiac index change from day 1 to day 0 in survivors and nonsurvivors. Cut-off at 0 L/min/m^2^.

In univariate analysis, a decrease in CCR-related parameters was associated with 28-day mortality (CI: −0.55 ± 0.73  *versus*  +0.18 ± 0.86 L/min/m^2^; *P* = 0.004; (Figure 3), CPI: −0.08 ± 0.12+  *versus*  +0.06 ± 0.16 W/m^2^; *P* = 0.005, DP: −2361 ± 3422  *versus*  −22 ± 3446 mmHg·bpm; *P* = 0.029).

In multivariate analysis (Figure 2), 28-day mortality was independently associated with a decrease in CPI (OR 1.84 [1.18–2.87] per 0.1 W/m^2^; *P* = 0.008) and CI (OR 1.30 [1.08–1.56] per 0.5 L/min/m^2^; *P* = 0.007).

### 4.4. Survival Curves (Figure 4)

**Figure 4:**
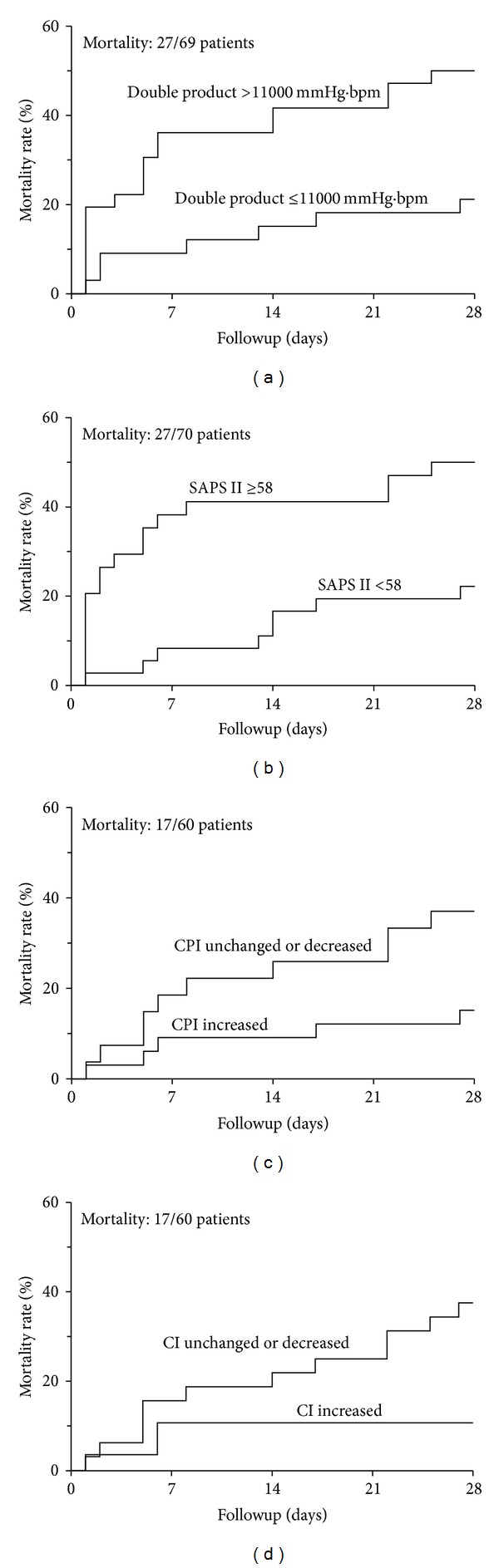
Kaplan-Meyer curves estimating the rate of death for a Double Product (DP) >11000 mmHg/s (a), a SAPS II >58 (b), a Cardiac Power Index (CPI) and a Cardiac Index (CI) either unchanged or decreased ((c) and (d)). bpm, beats per minute; SAPS II, Simplified Acute Physiology Score.

Figure 4 shows Kaplan Meier curves for all predicting significant factors from multivariate analyses: baseline DP >11000 mmHg·min and SAPS II >58; day-1 CI and CPI unchanged or decreased.

## 5. Discussion

The key finding of the present study is that in septic shock patients, without using any stress test, an early measurement (within the first 24 hours) of static and dynamic CCR parameters such as DP, CI, and CPI is independently associated with prognosis. 

### 5.1. Cardiac Contractile Reserve and Prognosis

The prognostic role of CCR parameters has been evaluated in different populations of critically ill patients. Tan and Littler estimated CCR in 28 cardiogenic shock patients by the response of the failing heart to incremental doses of dobutamine. A resting CI <1.3 L/min/m^2^, a cardiac power output ≤0.35 W, and a peak cardiac power output in response to dobutamine stress <1 W were clearly associated with poor outcome [11]. Data from the SHOCK trial registry demonstrated that, at inclusion, a low cardiac power output <0.53 W accurately predicted in-hospital mortality [5]. As expected, the mean value of CPI was extremely low at inclusion in cardiogenic shock (0.22 ± 0.08 W/m^2^ *versus*  0.62 ± 0.08 W/m^2^ in healthy subjects) while elevated in initial septic shock (0.8 ± 0.13 W/m^2^) [12, 13].

In severe sepsis and septic shock, the prognostic value of a positive response to dobutamine challenge has already been demonstrated in several studies using different indices, including oxygen delivery/oxygen consumption relationship and increase in cardiac and stroke volume indices [6, 14–17].

### 5.2. Initial and Spontaneous Evolution of CCR Parameters Is Related to Prognosis

In the present study, both endogenous and exogenous catecholamine stimulations were used to test CCR parameters without performing any superimposed pharmacological test. We found that, at inclusion, an elevated DP was significantly associated with death. Considering that SAP was not different between survivors and nonsurvivors, it could be hypothesized that deceased patients increased their HR in an attempt to compensate for a decreased stroke volume due to septic cardiomyopathy. However, HR could also have been increased as a result of other factors such as hemoglobin level (which was slightly different between survivors and nonsurvivors), acidosis, PaO_2_, and volemia. After twenty-four hours, we found that the decrease in CI and CPI predicted a higher mortality. Vieillard-Baron et al. also reported a decreased cardiac contractility after 48 hours of evolution in 34% of septic shock patients. Of note, cardiac index was also found to be decreased in nonsurvivors between admission and day one [2]. Indeed, these parameters are dependent on MAP, HR, and stroke volume, that is, to preload, afterload, and inotropism. In the present study, patients were resuscitated such as to obtain a relatively fixed MAP (65–75 mmHg). Therefore, all arterial pressure components (SAP, DAP, MAP, ISVR) were similar for survivors and nonsurvivors. Dobutamine was infused at very low doses; its administration was not different between survivors and nonsurvivors, and it is likely that its use did not influence the interpretation of the findings. Given this particularity, pressure components could be neglected and thus it would appear that patients that were not able to increase their CI displayed a worse prognosis, arguing for worse cardiac performance and inotropism. Similar findings have been reported by Werdan et al. who elegantly demonstrated, using a mathematical model, that it is possible to quantify the severity of septic cardiomyopathy by calculating the expected values of afterload-related CO/CI. Interestingly, these authors found a strong correlation between the intensity of septic cardiomyopathy and prognosis [3]. Of note, in the present study, CCR parameters were associated with prognosis in multivariate analysis while factors related to static cardiac function such as ejection fraction or related to vasoplegia (norepinephrine doses, ISVR) were not. Thus, the severity of septic cardiomyopathy was closely related to prognosis in septic shock. Whether this is a cause or consequence remains to be elucidated.

### 5.3. Study Limitations

This is a pilot, monocentric study with no validation cohort. Nevertheless, the most recent recommendations were followed to reduce the monocenter effect, and all our patients benefited from high level hemodynamic monitoring (ScVO_2_, lactate, PICCO, echocardiography). We made an effort to optimize the volemic status with a strategy that included fluid responsiveness prediction tests. However, this remained a genuine challenge, and we could not certify that preload dependency was not fully ruled out. Secondly, although we were able to identify patients with a high risk of death likely related to an inadequate CCR, we are currently unable to propose any demonstrated therapy to improve CCR during septic shock. In particular, dobutamine may be inefficient due to downregulation of myocardial *β*1-adrenoreceptors and may increase myocardial oxygen consumption [18, 19].

### 5.4. Conclusions

The present study adds significant knowledge regarding the importance of CCR during septic shock. Our results suggest that both CCR at inclusion, assessed using double product, as well as 24-hour CCR are important factors in determining patient prognosis. Importantly, in a therapeutic strategy in which arterial pressure is maintained to preestablished levels using norepinephrine, CCR parameters appear to be more predictive than vasoplegia-related parameters. Further studies are needed to determine the best therapeutic strategy in patients with decreased CCR.

## Key Messages

1. In septic shock patients, contractile cardiac reserve can be easily assessed with a nonpharmacological test.
2. At a fixed mean arterial pressure, a high double product at inclusion and a decrease in cardiac index and cardiac power index in the first 24 hours are related to poor outcome.

## Supplementary Material

Supplementary material contains in a first table the respiratory and biology description at inclusion and day 1. A second Table provided the sensitivity, specificity and area under ROC curves of the four factors associated with death at day 28.

## Figures and Tables

**Table 1:** Comparison of preexisting conditions, bacterial source, site of infection, and ICU evolution.

	28-day survival (*N* = 43)	28-day death (*N* = 27)	*P*
	m ± SD or *n* (%)	m ± SD or *n* (%)	
Age (years)	63 ± 17	62 ± 14	NS
Male gender	31 (72%)	19 (70%)	NS
Body mass index (kg/m^2^)	26.3 ± 7.0	27.2 ± 6.8	NS
Duration of hospitalization in ICU (days)	14.0 ± 12.4	9.8 ± 10.1	—
Preexisting conditions			
Diabetes	18 (42%)	3 (11%)	0.007
Denutrition	5 (12%)	4 (15%)	NS
Hypertension	24 (56%)	15 (56%)	NS
Ischemic heart disease	10 (23%)	4 (15%)	NS
Congestive heart failure	6 (14%)	3 (11%)	NS
Chronic renal failure	4 (9%)	4 (15%)	NS
Chronic respiratory disease	11 (26%)	6 (22%)	NS
Homeopathy and cancer	5 (12%)	7 (26%)	NS
Immunosuppression	10 (23%)	11 (41%)	NS
Bacterial source			
Gram-negative	7 (16%)	10 (37%)	0.030
Gram-positive	17 (40%)	8 (30%)	
Other*	3 (7%)	5 (19%)	
No pathogen	17 (40%)	4 (15%)	
Site of infection			
Lung	25 (36%)	18 (26%)	NS
Catheter	1 (1%)	3 (4%)	
Endocardium	4 (6%)	0	
Abdomen	6 (9%)	1 (1%)	
Soft tissues	1 (1%)	3 (4%)	
Kidney	5 (7%)	2 (3%)	

*Other: 2 *Pneumocystis Jirovecii*, 1 *Geotrichum *spp. ICU: intensive care unit.

**Table 2:** Hemodynamic characteristics at day 0 and day 1 in survivors and non-survivors.

	28-day survival				28-day death				P*	P*
	*n*	Day 0	*n*	Day 1	*n*	Day 0	*n*	Day 1	Day 0	Day 1
Volume expansion before inclusion (mL)	43	2326 ± 1843			27	2319 ± 1319			0.64	
Systolic blood pressure (mmHg)	43	114 ± 16	43	120 ± 15	27	119 ± 14	17	112 ± 15	0.24	0.083
Diastolic blood pressure (mmHg)	43	51 ± 9	43	54 ± 9	27	51 ± 8	17	52 ± 7	0.63	0.41
Mean arterial pressure (mmHg)	43	71 ± 9	43	75 ± 8	27	69 ± 5	17	72 ± 6	0.48	0.20
Heart rate (bpm)	43	97 ± 23	43	95 ± 23	25	115 ± 26	17	95 ± 20	0.007	0.97
Stroke Volume (mL)	43	34.6 ± 12.3	43	37.53 ± 10.1	27	35.3 ± 11.7	17	34.09 ± 11.7	0.50	0.34
Cardiac index (L/min/m^2^)	43	3.28 ± 1.18	43	3.46 ± 0.99	27	3.91 ± 1.35	17	3.03 ± 0.67	0.036	0.18
Systemic vascular resistance index (dyn·s/cm^5^·m^2^)	41	1660 ± 684	43	1669 ± 410	24	1435 ± 517	16	1702 ± 451	0.26	0.89
Global ejection fraction (%)	38	20 ± 7	40	21 ± 7	25	18 ± 7	15	17 ± 6	0.33	0.094
Left ventricle ejection fraction (%)	43	51 ± 12			27	55 ± 8			0.41	
Central venous oxygen saturation (ScvO2, %)	35	73 ± 11	34	72 ± 10	20	75 ± 11	13	72 ± 16	0.21	0.20
Central Venous Pressure (mmHg)	41	10 ± 5	43	9 ± 4	24	9 ± 5	16	9 ± 4	0.09	0.80
Global end diastolic volume (mL/m^2^)	42	785 ± 261	42	800 ± 251	27	835 ± 266	17	815 ± 299	0.37	0.76
Double Product (mmHg·bpm)	42	10990 ± 2847	42	10968 ± 2858	27	13310 ± 3385	17	10285 ± 2181	0.006	0.39
Cardiac power index (W/m^2^)	43	0.52 ± 0.21	43	0.58 ± 0.18	27	0.61 ± 0.22	26	0.48 ± 0.12	0.058	0.063
SOFA score	43	12 ± 3			27	12 ± 3			0.26	
SAPS II score	43	53 ± 16			27	69 ± 22			0.005	
Norepinephrine (*µ*g/kg/min)	43	0.63 ± 0.52	43	0.56 ± 0.79	27	0.95 ± 1.04	17	0.72 ± 0.61	0.33	0.13
Dobutamine (*µ*g/kg/min)			12	9.4 ± 5.8			5	7.4 ± 4.33		0.81
Duration of catecholamine administration (days)	43	5.2 ± 3.4			27	6.2 ± 7.5				
Lactatemia (mmol·L^−1^)	43	3.1 ± 3.0	40	2.5 ± 2	27	3.2 ± 2.6	13	2.1 ± 1.1	0.57	0.77

SOFA: Sequential Organ Failure Assessment; SAPS II: Simplified Acute Physiology *Score*. *P values from comparisons between survivors and non-survivors at day 0 and day 1 using the Mann-Whitney test.

## Abbreviations

CCR: Cardiac contractile reserve
CI: Cardiac index
CPI: Cardiac power index
CVP: Central venous pressure
DAP: Diastolic arterial pressure
DP: Double product
GEDV: Global end diastolic volume
HR: Heart rate
ISVR: Indexed systemic vascular resistance
LEF: Left ejection fraction
MAP: Mean arterial pressure
OR: Odds ratio
PP: Pulse pressure
SAP: Systolic arterial pressure
SAPS II: Simplified acute physiology score II
ScVO_2_: Central venous oxygen saturation
SOFA: Sequential organ failure assessment.
