# Supplementary material for: Cardiac Contractile Reserve Parameters Are Related to Prognosis in Septic Shock
Source: Biomed Res Int. 2013 Jul 17;2013:930673. doi: 10.1155/2013/930673 (PMC3730160; doi:10.1155/2013/930673)
Supplement: Supplementary file 1 — Supplementary material contains in a first table the respiratory and biology description at inclusion and day 1. A second Table provided the sensitivity, specificity and area under ROC curves of the four factors associated with death at day 28. [file 930673.f1.pdf]

Table : Respiratory and biology description at day 0 and day 1.* p-values from comparisons between survivors and non-survivors at Day 0 and Day 1 using the Mann-Whitney test.

	28-day survival				28-day death				P*	P*
	n	Day 0	n	Day 1	n	Day 0	n	Day 1	Day 0	Day 1
**Respiratory**										0.50
Inspired oxygen fraction (%)	43	85 ± 24	41	55 ± 20	27	79 ± 27	13	60 ± 22	0.21	0.52
Positive end-expiratory pressure (cm H_2O)	39	8 ± 3	37	7 ± 3	23	7 ± 3	13	7 ± 4	0.39	0.30
pH	43	7.34 ± 0.09	41	7.38 ± 0.10	27	7.27 ± 0.14	14	7.34 ± 0.11	0.022	0.19
pO_2 (mmHg)	43	161 ± 126	41	115 ± 42	27	124 ± 70	14	111 ± 36	0.66	0.97
pCO_2 (mmHg)	43	38 ± 9	41	37 ± 7	27	42 ± 14	14	37 ± 7	0.51	0.79
Lactate (mmol/l)	43	3.1 ± 3.0	40	3.8 ± 6.2	27	3.2 ± 2.6	13	2.1 ± 1.1	0.58	0.68
Bicarbonate (mmol/l)	43	21 ± 7	41	20 ± 6	26	18 ± 5	13	20 ± 4	0.054	0.50
Duration of controlled mechanical ventilation (days)	42	9.9 ± 8.5			27	8.0 ± 8.2			-	
**Biology**										
Creatinine (mg/l)	43	21.5 ± 17.1	41	15.7 ± 9.7	27	19.7 ± 11.6	13	19.5 ± 12.8	0.99	0.30
White blood cells (G/l)	43	13.5 ± 10.6	41	17.5 ± 15.1	27	9.8 ± 11.3	13	11.6 ± 12.8	0.067	0,12
Hemoglobin (g/dl)	43	10.5 ± 1.8	41	10.6 ± 1.4	27	9.2 ± 1.7	13	9.9 ± 1.9	0.005	0,22
Platelets (G/l)	42	241 ± 170	41	211 ± 123	27	152 ± 158	13	132 ± 108	0.010	0,04
Prothrombin ratio (%)	41	54 ± 18	32	53 ± 17	26	52 ± 15	8	59 ± 12	0,94	0,46
Troponin (µg/l)	43	0.69 ± 2.59	34	1.03 ± 1.85	25	0.47 ± 1.13	12	0.76 ± 1.80	0.79	0,25
B-type natriuretic peptide (ng/l)	29	625 ± 677	21	572 ± 709	19	934 ± 778	11	796 ± 797	0.12	0,48
Procalcitonin (mg/l)	36	34.1 ± 75.5	10	43.7 ± 83.3	24	29.4 ± 42.6	7	38.0 ± 49.5	0.24	0,42

Table : Association of significant factors with death at day 28. The sensitivity, specificity and area under ROC curves of the four factors illustrated in figure 4 were:

(AU ROC, Area Under the Receiver Operating Characteristic curve; SAPS II, Simplified Acute Physiology Score; CPI, Cardiac Power Index; CI, Cardiac Index.)

Factor	Cutoff	Sensitivity	Specificity	AU ROC
Double product	> 11000 mmHg.bpm	70%	60%	0.65
SAPS II	≥ 58	70%	65%	0.68
CPI decrease at day 1	≥ 0.0 W/m²	82%	65%	0.68
CI decrease at day 1	≥ 0 ml/min/m²	82%	58%	0.70
